# Extended Pleurectomy/Decortication for Malignant Pleural Mesothelioma: Humanitas’s Experience

**DOI:** 10.3390/jcm10214968

**Published:** 2021-10-26

**Authors:** Giuseppe Mangiameli, Edoardo Bottoni, Emanuele Voulaz, Umberto Cariboni, Alberto Testori, Alessandro Crepaldi, Veronica Maria Giudici, Emanuela Morenghi, Marco Alloisio

**Affiliations:** 1 Division of Thoracic Surgery, IRCCS Humanitas Research Hospital, Via Manzoni 56, 20089 Rozzano, MI, Italy; edoardo.bottoni@humanitas.it (E.B.); emanuele.voulaz@humanitas.it (E.V.); umberto.cariboni@humanitas.it (U.C.); alberto.testori@humanitas.it (A.T.); alessandro.crepaldi@humanitas.it (A.C.); veronica.giudici@humanitas.it (V.M.G.); marco.alloisio@humanitas.it (M.A.); 2 Department of Biomedical Sciences, Humanitas University, Via Rita Levi Montalcini 4, 20090 Pieve Emanuele, MI, Italy; 3 Biostatistic Unit, IRCCS Humanitas Research Hospital, Via Manzoni 56, 20089 Rozzano, MI, Italy; emanuela.morenghi@humanitas.it

**Keywords:** malignant pleural mesothelioma, extended pleurectomy/decortication (eP/D), trimodality treatment

## Abstract

Background: We analysed a series of malignant pleural mesothelioma (MPM) patients who consecutively underwent extended Pleurectomy/Decortication (eP/D) in a centre with a high level of thoracic surgery experience (IRCCS Humanitas Research Hospital) to explore postoperative morbidity and mortality, pattern of recurrence and survival. Methods: A retrospective analysis was performed on MPM patients underwent eP/D in our centre from 2010 to 2021. All patients were identified from our departmental database. Postoperative complications were scored according to Clavien–Dindo criteria. Survival analysis was performed by the Kaplan–Meier methods and Cox multivariable analysis. Results: Eighty-five patients underwent extended pleurectomy decortication (eP/D) during study period. Macroscopical residual disease (R2) was reported in one case. A neoadjuvant chemotherapy regiment was administrated in 88% of the surgical cohort. A complete trimodality treatment including induction with platinum agents and pemetrexed, radical cytoreductive surgery and volumetric modulated arc therapy technology (VMAT) could be administered in 63 patients (74%). Postoperative morbidity rate was 54.11%, major complications (defined as Clavien–Dindo ≥ 3) were reported in 11 patients (12.9%). Thirty-day mortality and 90-day mortality were, respectively, 2.35% and 3.53%. Median disease-free and overall survival were, respectively, 13.7 and 25.5 months. The occurrence of major complications (Clavien–Dindo ≥ 3), operative time, pT3–T4, pathological node involvement (pN+) were prognostic factors associated with worse survival. Conclusions: In our experience, eP/D is a well-tolerated procedure with acceptable mortality and morbidity, allowing for the administration of trimodality regimens in most patients. eP/D offered in a multimodality treatment setting have satisfactory long term oncological results. To obtain best oncological results the goal of surgery should be macroscopic complete resection in carefully selected patients (clinical N0).

## 1. Introduction

Malignant pleural mesothelioma (MPM) is a relatively rare a but highly malignant disease usually related to asbestos exposure [1]. MPM is associated with an extremely poor prognosis considering that a median survival of less than 1 year and a 5-year survival rate of less than 10% are commonly reported [2]. Recently, this tumour has been the subject of an increased scientific interest because its incidence has dramatically increased in high-income countries in the last years, as predicted by various prediction models previously developed [3].

In selected patients (good performance status and early-stage disease), “radical surgical procedures” have been offered in the past, with or without different induction/adjuvant treatments [4]. In 1996, Sugarbaker et al. have published a large series of MPM patients treated with extrapleural pneumonectomy (EPP) with a 5-year survival rate upper than 30% [5]. Similarly, in 2015, Lang-Lazdunski reported a 30.7% 5-year survival in MPM patients submitted to pleurectomy/decortication (P/D) as a multimodality therapy [6]. Interestingly a wide variability in EPP perioperative mortality has been reported ranging from 3 to 5% of Sugabaker series [7] to 18% of the MARS study [8].

Considering that MPM have a median of only a few months, perioperative outcomes—specifically mortality—should be carefully considered against the potential oncological benefit deriving from an aggressive surgery. Based on this consideration there is general consensus that in fact P/D is a less morbid option for certain eligible patients and may in fact lead to similar outcomes [9]. It is not by chance that today, National Comprehensive Cancer Network guidelines (v 2.2020) suggest P/D as surgery potentially safer than EPP that for early-stage MPM with epithelioid histology [10].

In our institution, we have adopted this scientific evidence migrating progressively from EPP (78 procedures between 2000 and 2010) to extended P/D (85 procedures since 2010). The aim of our study was to review our institutional surgical outcomes after eP/D to explore, postoperative morbidity and mortality, pattern of recurrence and survival. We also attempted to identify MPM patients who could potentially achieve the best oncological benefit from eP/D.

## 2. Materials and Methods

### 2.1. Patient Selection

A retrospective review was performed on MPM patients referred to the Humanitas Research Hospital, Milan, Italy, from 2010 to Avril 2021 in whom radical cytoreductive surgery was attempted. Only patients underwent cytoreductive surgery through eP/D, having 18 years or older and diagnosed with MPM, were enrolled in this study. Patients who underwent surgery without an attempt to perform maximal cytoreduction were excluded from this study. The American Joint Committee on Cancer Eighth Edition 7 was used to define pathological staging. Final pathological specimens were used to classify epithelioid, biphasic, or sarcomatoid histology. This work was performed with the approval of the Humanitas Research Hospital Internal Review Board.

### 2.2. Management Strategies

Our typical treatment strategy has been previously described [11]. Patient’s general status and cardiopulmonary reserve were systematically checked during pretreatment evaluation. All patients were staged with contrast-enhanced CT of the chest and abdomen, and FDG-PET scan. Magnetic resonance imaging was also used in case of suspicion of involvement beyond the pleural envelop [12,13]. Histological diagnosis was routinely obtained by video-assisted thoracoscopic surgery or CT-scan biopsy.

All cases were discussed during multidisciplinary team meetings. Broadly, clinical N0 patients with no evidence of chest wall invasion, mediastinal involvement or distant metastasis, epithelial histology, WHO performance status 0–1 and adequate cardiorespiratory reserve were considered appropriate candidates for surgery (predicted postoperative FEV1 > 1 L or >40% and left ventricular ejection fraction greater than 45%).

An induction regimen consisting of platinum-based regimen (cis-platinum 75 mg/m^2^ or carbo-platinum AUC 5) and pemetexed (PEM) 500 mg/m^2^ × 3/4 cycles has been consistently offered to all potential surgical candidates. 

The response to induction chemotherapy was evaluated through repetition of imaging studies, FDG-PET and ad hoc investigation by MRI, endosonography or mediastinoscopy according to m-RECIST criteria [14]. Deterioration of clinical conditions or disease progression after induction were exclusion criteria for surgery. A posterolateral thoracotomy in the VI intercostal space was our standard surgical approach. P/D and extended P/D always required complete removal of the visceral pleura from the entire lung surface, including macroscopically normal portions in the fissures down to the pulmonary artery. Extended P/D was performed in the case of macroscopic involvement of the pericardium or diaphragm. In such a case, bovine pericardium patch was systematically adopted for pericardium reconstruction. Macroscopically, normal parts of the diaphragm were spared as much as possible to facilitate primary reconstruction whenever possible. Otherwise, a Proceed^®^ (Ethicon, Johnson&Johnson, Somerville, NJ, USA) or bovine pericardium patch was used for diaphragm reconstruction. Hilar lymph nodes and at least two mediastinal stations were systematically harvested during P/D. After resection, patients were considered for adjuvant radiotherapy by volumetric modulated arc therapy technology (VMAT).

Postoperative follow-up was carried out by 3–6 monthly clinical assessment, CT scans of the chest and ad hoc further testing. Follow-up data were also obtained by contact with families and general practitioners, from hospital charts and health registries. The follow-up closing date was 1 July 2021. The postoperative complications were scored according to Clavien–Dido criteria [15].

### 2.3. Outcomes and Statistical Analysis

Disease-free survival (DFS) and overall survival (OS) were the primary outcomes of this study. OS was measured from the day of surgery until death from any cause or last contact. Disease-free survival (DFS) was measured from the day of surgery until first recurrence. Perioperative mortality was defined as death within 30 days of surgery or the same hospital stay. We further verified perioperative mortality by manually reviewing patient records for causes of death. Categorical data were described as number and percentage; for quantitative data median and interquartile range (IQR), median and range, or mean and standard deviation was used as appropriate. Owing to low numbers in each p-T and p-N subcategory, groups including p-T 0-1-2 and p-N 0 were built, and p-T 3-4 and p-N+ were left as categorical variables. Survival analysis was performed by the method of Kaplan–Meier, with observation times censored to the date of last contact for patients who were still alive. Survival comparisons between groups were performed by the Cox regression method, including in the multivariate model only those parameters reaching a value of *p* ≤ 0.1 in the univariate analysis. A significance level of 5% (*p* < 0.05) was adopted for comparisons. For all analyses, the Stata software (V.13, StataCorp LLC, College Station, TX, USA) was used. 

## 3. Results

We identified 85 patients who underwent eP/D with radical intent, no palliative and biopsy procedures were included in this analysis. Only one macroscopically incomplete P/D was carried out, due to superior vena cava invasion. The potential median follow-up time, defined as time from surgery to 1st July 2021 for all patients, was 39.3 months [19.8–77.8]. Baseline characteristics of patients are summarized in Table 1.

Diagnosis of MPM was more frequently obtained through thoracoscopy (80/85 patients), and a chemical pleurodesis was simultaneously performed during thoracoscopic exploration in 88% of cases (*n* = 75). Epithelioid histology was the most common (88% of cases), a biphasic histology was found in four patients (4.7%). Any kind of chemotherapy and/or radiotherapy (both in the neoadjuvant and adjuvant settings) was administered to 81 patients (95%). A complete trimodality treatment including induction with platinum agents and PEM, radical cytoreductive surgery and VMAT could be administered in 63 patients (74%).

A pathological complete respond (no evidence of residual disease) after induction treatment was identified in three patients (3.5%), 44 patients (51.7%) had a pathologically confirmed stage I MPM. In 37.6% of cases a nodal involvement (pN+) was present at final pathological examination. 

Mean operative time was 380 ± 73 min. A diaphragmatic resection was performed in 83 patients (97.64%), it was associated with pericardium resection due to macroscopic involvement of both in 34 patients (40%). The resected pericardium was systematically replaced with bovine pericardium for the reconstruction, diaphragm reconstruction was performed by directed suture in 38 (44.7%) patients. In the remaining cases (*n*= 45), a polypropylene mesh (*n* = 39) was the more frequently choice followed from bovine pericardium (*n*= 6) for diaphragmatic reconstruction.

The early postoperative course was uneventful in 39 (45.8%) patients, whereas minor only (Clavien–Dindo ≤ 3) or major complications (Clavien–Dindo > 3) occurred respectively in 35 (48.2%) and 11 (12.9%) cases. Prolonged air leak was both the most common postoperative complication (21/46) and the most common minor complication (Clavien–Dindo < 3) occurring in 16 out of 35 minor complications. Empyema was the most common major complications (Clavien–Dindo ≥ 3) occurring in 5 out of 11 complicated patients. The 30- and 90-day early mortality rates were 2.35% and 3.53%, respectively. Mean hospital stay was 15 (range: 7–70) days.

Median follow-up for the whole series was 16.2 months (range: 0.7–125.3). Follow-up and survival data of all patients are reported in Table 2. 

At that time, 24 patients (28.23%) were still alive and disease-free, 16 (18.82%) were alive with disease, and 45 (52.94%) had died. Mesothelioma recurrence or progression was the cause of death in 39 patients (45.88%), six (7.05%) patients had died of other causes. The most common pattern of recurrence was combined local and distant presented in 35 patients (62.5%) (See Table 2). 

**Figure 1:**
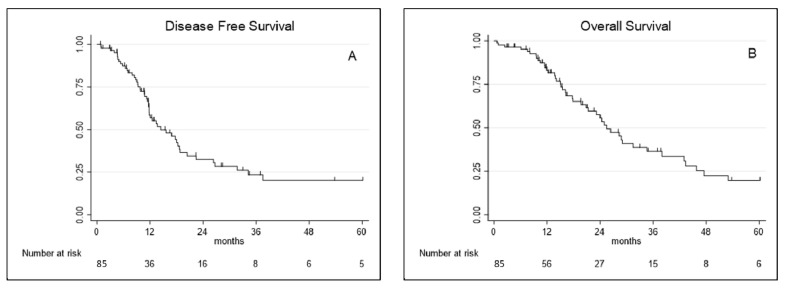
(**A**) Disease-free survival and (**B**) overall survival curves for extended pleurectomy decortication (eP/D).

Median disease-free survival and overall survival were, respectively, 13.7 months (95% CI 9.01–31.71) and 25.5 months (95% CI 15.07–47.5). DFS and OS probability (%) at 1, 3 and 5 years are reported in Table 2 and showed in Figure 1.

All clinical and biological characteristics investigated in cox regression analysis of DFS and OS are reported, respectively, in Table 3 and Table 4. 

**Figure 2:**
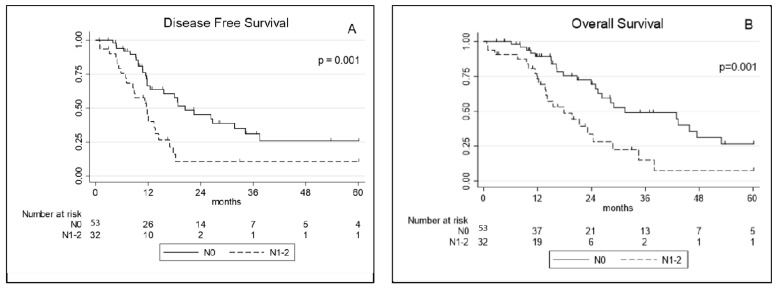
(**A**) Disease-free survival and (**B**) overall survival curves for extended pleurectomy decortication (eP/D) according to pathological node involvement (pN0 vs. pN+). N0: no nodes involvement, N1–2: nodes involvement (N+).

At univariate analysis, RDW, the occurrence of major complications (Clavien–Dindo > 3), operation time, a pT3–4 and pathological node involvement (pN+) were significantly associated with worse DFS. At multivariate analysis operation time and pN+ were the only factors significantly associated with worse DFS (Table 3). Concerning OS, the occurrence of major complications (Clavien–Dindo > 3), operation time, a pT3–4, and pN+ were prognostic factors significantly associated with reduced survival at univariate analysis. At multivariate analysis the occurrence of major complications (Clavien–Dindo > 3), operation time and pN+ retained a significant association with worse OS (Table 4; Figure 2). 

## 4. Discussion

The purpose of this study was to evaluate comorbidity, postoperative morbidity, and survival in patients undergoing radical cytoreductive surgery for MPM in a high-volume centre that adopted extended pleurectomy-decortication (eP/D) instead of extrapleural pneumonectomy (EPP) as main surgical approach in a multimodality setting. We, as many surgical groups, changed our surgical approach to MPM in September 2010 as a consequence of the increasing scientific evidence that questioned the benefit of EPP in terms of survival extension [16,17]. In our experience, this change was justified from a reported lower short-term mortality than EPP [9], allowing us to extend surgical indication to older patients. 

The main characteristics of our patients are comparable with those of other similar retrospective studies (Table 5) as to mean age, sex, and pathological stage [16,18,19,20,21,22,23,24,25,26]. 

In our series, there was a slightly lower prevalence of stage III–IV disease in comparison to the studies published until 2014 which report percentage ranging from 63 to 100%. We have reported 46% of stage III and no stage IV, but it is difficult to tell whether the slightly higher percentage of stage I–II disease in our eP/D series reflects patient selection (clinical N+ patients were usually excluded from surgery) or the effect of the systematic administration of preoperative chemotherapy with platinum-pemetrexed in recent years. 

Perioperative complications occurred in 46 patients (54%) and were grade 3+ in 11 (12.9%). Interestingly the rate of grade 3+ was lower (12.9 vs 27%) compared with our initial surgical experience in MPM by performing EPP reported in a recent article [11] confirming that P/D is a less morbid procedure than EPP and supporting our surgical shift from EPP to P/D. As consequence of systematic peeling of visceral pleura the most common complication was prolonged air leak, resulting in a median hospitalization stay of 15 days. Instead, the most common major complication was empyema that occurred in five patients of whom four had submitted to diaphragm reconstruction by a mesh graft. For this reason, in our surgical strategy, we systematically tried to spare the diaphragm and close it primarily in order to avoid the risk of graft infection [27]. Finally, our early postoperative complication rates and 30- and 90-day mortality rates of 2 and 3% are similar to the other published P/D series (Table 5). 

Several perioperative techniques and strategies have been adopted from surgical teams to improve survival in MPM patients. Multimodality strategies, proposing surgery, chemotherapy, and radiotherapy combined in various orders, are shown to improve survival with reported median survivals between 17 and 35 months and 5-year survival of 15% to 20% in different series [17,28,29,30,31].

In several multimodality therapy trials with EPP [32,33,34] only 50–62% of eligible patients were able to tolerate the full treatment regimen. In a recent study, where we compared our initial experience with P/D vs. EPP in treating MPM, this evidence was confirmed, considering that only 31% of our patients have been able to receive a trimodality treatment after EPP [11]. 

In our series as well, 74% of the patients could receive a full trimodality course. Probably, the impact of eP/D on patients’ general condition was apparently less severe as most patients are likely to complete trimodality regimens [20]. Interestingly, despite the lung was still in place, we did not observe any severe complications of radiotherapy. We have exclusively observed one case of grade 2 radiation pneumonia but no grade 3 or higher complications after VMAT.

In our early experience, overall and disease-free median survival data are comparable with the recent literature data of P/D series. We have finally reported a median OS of 25. 5 months and a median DFS of 13.7 months. 

In the present study of 56 patients who had recurrence after eP/D, 11 (19.6%) showed local recurrence, four (7.1%) showed distant recurrence, and 35 (62.5) showed both local and distant recurrences. Our results are in contrast with a recent series in which P/D was associated with a larger proportion of local recurrence (68.4%) [24]. Our results can probably be explained by the high percentage of patients submitted to VMAT after surgery (74%) as well as by careful selection of patients (pathological stage I+II was noted in 54% of patients and histological assessment revealed the final pathology as epithelioid in 88.2% of patients). 

Several prognostic factors that we could explore were associated with better overall and disease-free survival. Interestingly, univariate analysis disclosed preoperative RDW as strictly related to disease-free survival (*p* = 0.04), but it was not confirmed in the exploratory multivariable analysis where it was however associated to *p* close to significance (*p* = 0.053). These preliminary data are interesting considering that RDW (a measure of the variation of erythrocyte volume) has recently been advocated as a prognostic tool in neoplastic and non-neoplastic diseases. Particularly in a recent study pre-operative RDW was an effective prognostic factor of disease-free survival in resected pN1 lung adenocarcinoma [35]. Our results could be interesting in consideration that is the first evidence of a RDW as possible prognostic factor in a surgical series of MPM after a study that confirmed RDW as significant predictive factor for MM prognosis in not surgical series [36]. In our series, LNR and PLR were not associated with better overall and disease-free survival.

The occurrence of major complications was a prognostic factor having a negative impact on OS. These data confirm the results reported in our previous study carried out of all MPM patients operated in our institution from 2000 to 2015. In this study, the occurrence of grade 3+ complications was associated with OS independently of the type of surgery performed (P/D or EPP) [11]. In addition, in the current study, the operative time was a prognostic factor of both DFS and OS. As far as we know, it is the first time that this data is reported in literature about mesothelioma surgery while several studies have reported an association between operative time and occurrence of perioperative complications in lung and colorectal surgery [37,38]. We can speculate that the occurrence of complications associated to longer operative time is capable to influence survival outcomes in oncological patients. Finally, in our cohort, pathological nodes involvement (pN+) was one of strongest prognostic factors of DFS and OS as already reported by other authors [31,34,38]. The relatively lower frequency of patients with N+ status in our cohort of patients (37.6%) was probably secondary to avoidance of straightforward surgery for patients with clinical nodes disease. As for the N descriptors, in our survival analysis did not yield any difference between pN1 and pN2 by using the seven TNM classification [39]. Our data support the eighth TNM classification where only two N categories (N1 and N2) remain. This N category reclassification is due to the fact that intrapleural and extrapleural nodes are now grouped into category N1 because it seems that for MPM, survival is more affected by the number of nodes involved than by the specific anatomical locations of nodal disease as in lung cancer [40,41,42,43,44].

The main limitation of this study is its retrospectivity. The strengths of this study are that all patients were treated and followed in a single centre, surgical procedures were performed by experienced surgeons in a high volume centre with 30 years of experience in treating MPM. Probably for these reasons OS in our patient cohort was identical or higher than that reported in other P/D series [19,20,21,22,23,24,25,26].

## 5. Conclusions

In conclusion, our data support the concept that eP/D is a well-tolerated procedure with a slightly reduced mortality and morbidity, allowing for the administration of trimodality regimens in most patients. In our experience, eP/D offered in a multimodality treatment setting have satisfactory long term oncological results. In any case, the goal of surgical resection should be macroscopic complete resection in careful selected patients (clinical N0) and to return patients to potential oncologic therapy.

## Figures and Tables

**Table 1:** Patients’ clinical and pathological features.

Variables	Value (*n* = 85)	%
Mean age	65.2 (63.5–67.0)	
Male gender	62	72.94%
Mean BMI	22.7 (22.2–23.3)	
Smoker	44	51.76%
Asbestos exposure	75	88.23%
Any comorbidity	42	49.41%
DLCO% (*n* = 41)	71.3 (66.5–76.2)	
FEV1 %	79.6 (76.2–82.9)	
RDW	16.8 (16.2–17.4)	
NLR	2.98 (2.63–3.34)	
PLR	11.7 (10.2–13.2)	
Talc pleurodesis	75	88.24%
Right sided tumour	49	57.65%
Tumour histology		
Epithelioid	75	88.23%
Sarcomatoid	1	1.17%
Biphasic	4	4.70%
pT category		
T0–1	6	7.05%
T2	18	21.17%
T3	43	50.58%
T4	18	21.17%
pN category		
N0	53	62.35%
N1	18	21.18%
N2	14	16.47%
N+	32	37.65%
TNM stage		
I	44	51.76%
II	2	2.35%
III	39	45.88%
Chemotherapy		
Any induction	75	88.24%
Any adjuvant	4	4.76%
VMAT	66	77.64%
Trimodality therapy	63	74.11%
Median hospitalization (days)	15 (7–70)	
Clavien–Dindo	46	54.11%
<3	35	48.23%
≥3	11	12.94%
30-day mortality	2	2.35%
90-day mortality	3	3.53%

**Table 2:** Follow-up and survival data.

	eP/D (*n* = 85)	Range
Median follow-up (months)	16.2	0.7–125.3
Recurrence (*n*)	56 (66.67%)	%
Local	11	19.64%
Local + distant	35	62.50%
Distant	4	7.14%
Unknown	6	10.71%
Median survival (months)		**IQR**
Disease-free	13.7	9.01- 31.71
Overall	25.5	15.07–47.5
OS probability (%)	**%**	**95% CI**
1-year	83.07	72.57–89.82
3-year	36.48	23.74–49.31
5-year	19.64	9.27–32-84
DFS probability (%)	**%**	**95% CI**
1-year	56.16	44.05–66.63
3-year	18.93	9.74–30.43
5-year	16.22	7.58–27.75

**Table 3:** Details of the statistical analysis: univariate and multivariate analyses—disease-free survival (Cox proportional hazards model).

DFS	Univariable			Multivariable		
Variable	HR	95% (CI)	*p*-Value	HR	95% (CI)	*p*-Value
Age	0.99	0.96–1.02	0.572			
Male sex	0.81	0.46–1.43	0.476			
BMI	1.05	0.95–1.16	0.330			
Any comorbidity	0.63	0.36–1.08	0.093	-		
Smoke	1.45	0.56–3.74	0.438			
Asbestos exposure	0.79	0.34–1.87	0.594			
FEV1 %	1.00	0.98–1.02	0.989			
DLCO%	1.01	0.98–1.03	0.689			
RDW	1.08	1.00–1.17	0.042	-		
NLR	0.89	0.74–1.07	0.213			
PLR	1.00	0.95–1.04	0.952			
Induction therapy	0.90	0.41–1.99	0.794			
Trimodality therapy	0.59	0.32–1.07	0.083	-		
Operation time	1.32	1.07–1.63	0.010	1.36	1.10–1.68	0.001
Right side	1.10	0.65–1.87	0.731			
Any complication	1.49	0.86–2.59	0.153	-		
C-D complication ≥3	2.36	1.03–5.41	0.043	-		
pT3–4	2.99	1.52–5.86	0.001	-		
pN+	2.53	1.46–4.37	0.010	2.58	1.47–4.53	0.004

**Table 4:** Details of the statistical analysis: univariate and multivariate analyses—overall survival (Cox proportional hazards model).

OS	Univariable			Multivariable		
Variable	HR	95% (CI)	*p*-Value	HR	95% (CI)	*p*-Value
Age	1.01	0.97–1.04	0.734			
Male sex	0.99	0.52–1.87	0.975			
BMI	1.08	0.96–1.21	0.207			
Any comorbidity	0.83	0.46–1.51	0.542			
Smoke	2.01	0.69–5.83	0.200	-		
Asbestos exposure	0.83	0.29–2.36	0.730			
FEV1 %	1.00	0.98–1.02	0.878			
DLCO%	1.00	0.96–1.03	0.827			
RDW	1.07	0.99–1.16	0.079	-		
NLR	0.86	0.69–1.08	0.197			
PLR	0.99	0.94–1.04	0.589			
Induction therapy	0.82	0.37–1.85	0.640			
Trimodality therapy	0.77	0.39–1.52	0.455			
Operation time (hour)	1.36	1.06–1.76	0.017	1.40	1.07–1.84	0.015
Right side	1.03	0.57–1.87	0.926			
Any complication	1.52	0.82–2.83	0.181			
C-D complication ≥3	1.04	1.31–7.02	0.009	2.80	1.20–6.53	0.017
pT3–4	1.97	1.01–3.86	0.047	-		
pN+	2.71	1.48–4.95	0.001	2.74	1.49–5.06	0.001

**Table 5:** Postoperative complications, mortality, and outcomes after pleurectomy-decortication.

Author	Year	*n*	Age	Males	Epithel.	Stage 3–4	R2	Grade 3	30-D	90-D	Median OS
Flores [16]	2009	278	63	220 (79)	178 (64)	180 (65)	-	25 (9)	13 (5)	-	12
Nakas [18]	2012	67	61	-	67 (100)	67 (100)	2 (3)	10 (15)	2 (3)	8 (12)	13,4
Lang-Ladz. [19]	2012	54	63	47 (87)	36 (67)	34 (63)	3 (6)	-	0 (0)	-	23
Bolukbas [20]	2013	88	66	73 (83)	70 (80)	57 (65)	31 (35)	-	2 (2)	-	26,3
Burt [21]	2014	130	68	104 (80)	-	-	-	6 (5)	4 (3)	-	-
Nakas [22]	2014	140	59	122 (87)	86 (61)	-	-	-	-	-	16,2
Bovolato [23]	2014	202	63	149 (74)	147 (77)	66 (33)	-	-	(2.6)	(6)	20.5
Batirel [24]	2016	130	56	76 (58)	95 (75)	-	60 (46)	17 (13)	6 (4.6)	13 (10)	17.8
Nakamaura [25]	2020	90	66	78 (87)	85 (94)	26 (29)	4 (4)	-	-	-	57
Zhou [26]	2021	95	65	71 (75)	71 (75)	44 (46)	-	-	0 (0)	4 (4)	18
Humanitas (current study)	2021	85	65	(73)	80 (94)	39 (46)	1 (3)	11 (13)	2 (2)	3 (3)	25.5

## Data Availability

Data derives from our istitutional database.
